# Primary Bone Marrow B-Cell Lymphoma Undetected by Multiple Imaging Modalities That Initially Presented with Hypercalcemia

**DOI:** 10.1155/2018/7676580

**Published:** 2018-07-26

**Authors:** Jin Sae Yoo, Juwon Kim, Hyeong Ju Kwon, Jung Soo Lim

**Affiliations:** ^1^Department of Internal Medicine, Wonju Severance Christian Hospital, Yonsei University Wonju College of Medicine, Wonju, Republic of Korea; ^2^Department of Laboratory Medicine, Wonju Severance Christian Hospital, Yonsei University Wonju College of Medicine, Wonju, Republic of Korea; ^3^Department of Pathology, Wonju Severance Christian Hospital, Yonsei University Wonju College of Medicine, Wonju, Republic of Korea; ^4^Institute of Evidence Based Medicine, Wonju Severance Christian Hospital, Yonsei University Wonju College of Medicine, Wonju, Republic of Korea

## Abstract

**Purpose:**

We report a rare case of severe hypercalcemia that was ultimately diagnosed as primary bone marrow diffuse large B-cell lymphoma (BCL).

**Case Report:**

A 74-year-old male patient visited our hospital complaining of tenderness and swelling of the left knee caused by supracondylar fracture of the left distal femur. His initial blood tests showed a serum calcium level of 13.9 mg/dL, inorganic phosphorus of 4.34 mg/dL, and a serum creatinine level of 1.54 mg/dL. A serum assay of intact parathyroid hormone showed 5.24 pg/mL, and the patient's serum 25(OH)D level was 22.33 ng/mL. To exclude malignancy, we performed imaging studies, including abdomen or chest computed tomography and positron emission tomography-computed tomography; however, no suspicious lesion was found, although the serum PTH-related peptide level was elevated at 4.0 pmol/L. A bone marrow biopsy was performed to identify any hidden hematologic malignancy. As a result, the pathology of bone marrow confirmed the presence of atypical lymphocytes that stained positive for the CD20 marker, which is consistent with BCL involving the bone marrow.

**Conclusion:**

This case highlights the importance of pursuing a thorough workup for rare underlying causes of hypercalcemia when parathyroid-related etiologies can be excluded.

## 1. Introduction

Hypercalcemia is a frequent finding in patients presenting to both outpatient clinics and emergency departments. While mild hypercalcemia is usually asymptomatic, patients with more severe hypercalcemia may present with a wide range of symptoms and signs, ranging from nausea, lethargy, and altered consciousness to life-threatening renal failure and cardiac arrhythmia that may lead to mortality if left unmanaged [1, 2].

Common causes of hypercalcemia are parathyroid-related diseases and malignancy [1]; one study investigated patients who visited the emergency department of a single hospital for hypercalcemia and found that 44% of cases were caused by malignancies [3]. In addition, malignancy accompanied by hypercalcemia is usually associated with a poor prognosis. Therefore, an aggressive, thorough investigation into the etiology of hypercalcemia is warranted whenever it is encountered [1].

We report a case of a 74-year-old male patient who presented with hypercalcemia and was ultimately diagnosed with primary bone marrow diffuse large B-cell lymphoma (DLBCL).

## 2. Case Report

A 74-year-old male patient visited the emergency department of our hospital for tenderness and swelling of the left knee. He had a past history of hypertension, spinal stenosis, benign prostate hypertrophy, and unruptured aneurysm of the right carotid artery. He also had visited an orthopedic surgery clinic due to an old fracture of the right tibial tuberosity six weeks previously. Initial physical examination found tenderness and crepitus of the left knee. Radiographs of both knee joints confirmed the diagnosis of supracondylar fracture of the left distal femur. His initial vital signs were as follows: blood pressure 164/81 mmHg; heart rate: 66 beats per minute; respiratory rate: 18/min; and body temperature: 36.0°C.

The results of an initial complete blood cell count were within the normal range: white blood cell count 8.66x10^9^/L with 70.3% neutrophils, hemoglobin 14.1 g/dL, hematocrit 40.7%, and platelet count 173x10^9^/L. Serum blood urea nitrogen and creatinine were 15.2 mg/dL and 1.07 mg/dL, respectively. In addition, serum calcium and alkaline phosphatase levels were elevated at 13.9 mg/dL and 152 U/L, respectively; inorganic phosphorus and serum albumin levels were within the normal ranges (4.34 mg/dL and 3.9 g/dL, respectively). Thyroid function test was also normal. The patient underwent a successful closed reduction and retrograde intramedullary nailing of the fractured joint the following day. However, he soon began to complain of general weakness, nausea, vomiting, and anorexia. His serum creatinine level was increased to 2.37 mg/dL on postoperative day 7, while the hemoglobin level decreased to 8.2 g/dL on postoperative day 9. On postoperative day 10, the patient's right distal femur fractured when rising from a wheelchair. Whole-body radionuclide bone scan with technetium-99 found multiple focal activities at bilateral femoral shafts, both knee joints, and left tibia (data not shown).

He was referred to our endocrinology department for evaluation of hypercalcemia. A serum assay of intact parathyroid hormone (iPTH) was 5.24 pg/mL (range: 15-65), and the serum 25(OH)D level was 22.33 ng/mL. In addition, a workup for anemia was concurrently carried out: serum iron of 77 *μ*g/dL (range: 65-175), total iron binding capacity of 296 *μ*g/dL (range: 250-425), ferritin of 761.91 ng/mL (range: 22-322), and serum lactate dehydrogenase of 258 U/L (range: <290 U/L). The peripheral blood smear was unremarkable, except for moderate toxic changes in neutrophils. His reticulocyte index was 0.6%, which implied inadequate red blood cell production despite the presence of anemia. Also, *β*2-microglobulin was elevated to 7.33 mg/L (range: 0.81-2.19). Due to the combination of persistent hypercalcemia, renal failure, anemia, and bone lesions, serum and urine electrophoresis were performed to rule out the possibility of multiple myeloma, both of which were negative. However, serum PTH-related peptide (PTHrP) assay showed a level elevated to 4.0 pmol/L (range: <1.1).

**Figure 1:**
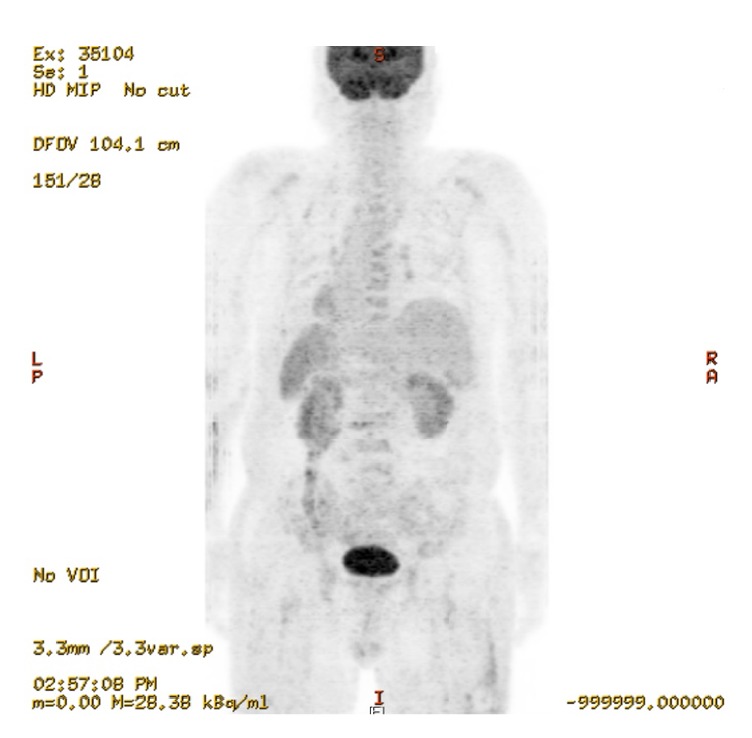
Torso positron emission tomography-computed tomography scan demonstrated no abnormal FDG uptake.

**Figure 2:**
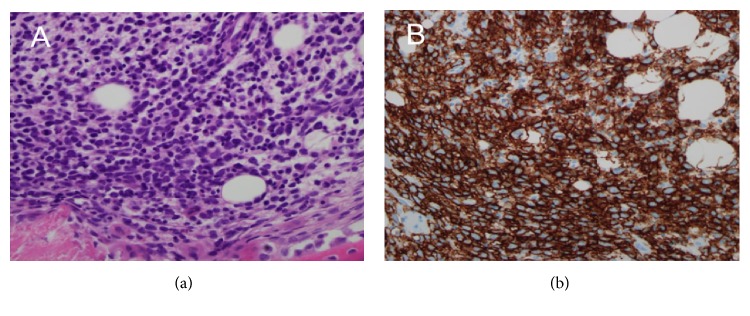
Histopathology of bone marrow biopsy specimen. (a) Bone marrow biopsy (hematoxylin-eosin stain, x200) shows infiltration of large atypical cells. (b) The immunohistochemical staining for CD20 (x100) is diffusely positive in large atypical cells.

**Figure 3:**
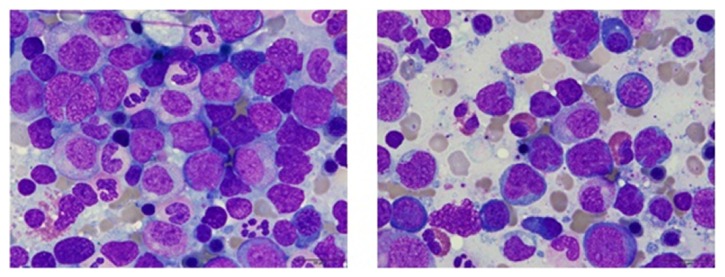
Bone marrow aspiration (Wright-Giemsa stain, x1000) shows infiltration of diffuse large B-cell lymphoma. Large, atypical cells with irregularly shaped nuclei and basophilic cytoplasm are noted in clusters.

The patient underwent a complete diagnostic workup to find any hidden malignancy. Serum tumor biomarkers were all within normal ranges (carcinoembryonic antigen: < 2 ng/mL, cancer antigen 19-9: 7.3 U/mL, alpha-fetoprotein: 3.71 ng/mL, and prostate specific antigen: 0.17 ng/mL). A thin-slice (1 mm) chest CT and abdominopelvic CT scans were unremarkable (data not shown). A torso positron emission tomography-computed tomography (PET-CT) scan also showed no abnormal FDG uptake (Figure 1). To exclude hematologic malignancy, a bone marrow biopsy at the left iliac crest was performed. The bone marrow smear revealed several large atypical lymphocytes with a high nucleus-to-cytoplasm ratio and prominent nucleoli. Initial chromosomal karyotyping revealed both aneuploidy and multiple deletions and translocations across a number of chromosomes, including 73 chromosomes, XXY, del(1)(p22), and (q21;q26.2). Pathology of the bone marrow confirmed the presence of atypical lymphocytes that stained positive for the CD20 marker, which is consistent with B-cell lymphoma (BCL) involving the bone marrow. Immunophenotyping of bone marrow cells further supported the diagnosis of BCL, with the population testing positive for CD45, CD19, CD20, CD79a, and HLA-DR (Figures 2 and 3); however, terminal deoxynucleotidyl transferase expression in bone marrow cells was negative. This case was confirmed to be a rare case of primary bone marrow DLBCL.

## 3. Discussion

This case emphasizes the importance of detecting less common causes of hypercalcemia if the clinical presentation of other causes of hypercalcemia is not definite when performing diagnostic workups. Besides primary hyperparathyroidism and malignancy, there are other numerous conditions associated with hypercalcemia: granulomatous diseases such as sarcoidosis and tuberculosis; drugs like lithium carbonate and thiazide diuretics; prolonged immobilization; endocrine disorders including thyrotoxicosis and adrenal insufficiency [1]. The first step for evaluating the cause of hypercalcemia is the measurement of iPTH; then, the possibility of cancer should be considered in the setting of low serum iPTH concentration [1]. PTHrP is often measured to screen for malignancy-induced hypercalcemia [1]; however, PTHrP elevation can be observed in various benign conditions such as systemic lupus erythematosus with multiple organ involvement [4]. Therefore, caution is necessary when interpreting laboratory or radiographic findings in patients with hypercalcemia who showed PTHrP elevation.

Several case reports on lymphomas that initially presented with severe, unexplained hypercalcemia have been published [5–7]. Ha et al. [5] reported a case of intravascular large BCL in a 68-year-old male who presented with lethargy and weight loss. Similar to our case, their patient also showed hypercalcemia with decreased serum iPTH and elevated PTHrP levels. However, his PET-CT scan demonstrated strong, diffuse uptake in the bones and spleen. Kapur and Levin [8] also described a 69-year-old female who presented with worsening confusion and weight loss; in their case, multiple large intra-abdominal and pelvic masses were visible on abdominopelvic CT and PET-CT scans. However, the malignancy-associated hypercalcemia described in our case was unique in that lesions suggestive of malignancy were not found across several imaging modalities.

Furthermore, primary bone marrow BCL is a very rare disease in itself. Bhagat et al. [9] described a case series of primary bone marrow lymphoma from a single tertiary care center, which reported a total of four cases over the past 15 years. The median age was 52 years, and chief complaints included general weakness, fatigue, and dyspnea on exertion. All patients presented with a moderate degree of anemia and showed hypercalcemia, as in our case. The prognosis was generally poor; two patients died while receiving standard chemotherapy, and two additional patients experienced a relapse despite achieving remission after completion of the first round of chemotherapy.

Unfortunately, no bone tissue was obtained during the surgery, because this patient was referred to our department for the workup for hypercalcemia after the surgery. In addition, the bone scintigraphy was performed 1 month after the surgery, and in such clinical setting, the focal activities may reflect postsurgical inflammation or healing process at and around the site of injury instead of malignancy. Recently, Zhang X et al. [10] demonstrated clinical characterization and outcome of 61 patients with primary bone lymphoma (PBL); their mean age was 45 years, and patients aged less than 60 years accounted for approximately 75%. Those with PBL commonly showed soft tissue invasion (68.9%), lymph node involvement (44.3%), and B symptoms (31.1%), and the most common sites in all patients with PBL were the spine and pelvic bones [10]. Furthermore, a majority of PBL patients had a localized disease rather than multifocal disease [11, 12].

In contrast, our patient was very much older than that of previous reports regarding PBL. He had no associated signs or symptoms, including nerve compression, local mass, soft tissue invasion, lymph node involvement, and B symptoms. Furthermore, there were no signs of hemophagocytosis, such as high fever, liver failure, or coagulopathy; clear evidence of intravascular lymphoma in the pathology was also not found. Moreover, the bone scintigraphy showed unremarkable skeletal activities in spine or pelvic bones, which are the most common sites of PBL. Particularly, ^18^F-PET-CT is known to play important roles in the decision making of diagnosis and treatment response as well as the determination of recurrence and residual disease in patients with PBL [13], because PBL is usually presented as a hypermetabolic lesion on FDG-PET [14]. In this case, the PET-CT demonstrated no abnormally hypermetabolic lesion throughout skeletal systems, which makes the possibility of PBL unlikely, even though this image did not show the entire body.

According to Stewart [15], the mechanisms behind hypercalcemia associated with cancer can be divided into four categories: (1) humoral hypercalcemia of malignancy (HHM), which accounts for approximately 80% of cases; (2) local osteoclastic hypercalcemia consisting of 20% of cases; (3) 1,25(OH)_2_-dihydroxyvitamin D (1,25(OH)_2_D) secreting lymphomas (<1%); and (4) ectopic hyperparathyroidism (<1%). HHM involves the overproduction of PTHrP by malignant tissues (most often squamous-cell cancer, breast cancer, and non-Hodgkin's lymphoma), which circulates throughout the body and ultimately stimulates bone resorption and calcium storage release [15]. Osteoclastic hypercalcemia is predominantly found in breast cancer and multiple myeloma due to extensive bone involvement of the malignancies [15]. Active secretion of 1,25(OH)_2_D by certain lymphomas enhances osteoclastic bone resorption and intestinal calcium absorption, causing hypercalcemia [15]. In this case, HHM appears to be the most likely mechanism behind the hypercalcemia as the PTHrP was elevated, although the possibility of 1,25(OH)_2_D secreting lymphoma could not be completely ruled out.

In conclusion, this rare case of primary bone marrow DLBCL suggests that hypercalcemia should not be overlooked. A thorough workup of rare underlying causes of hypercalcemia should always be considered when parathyroid-related causes can be excluded.

## Data Availability

The data used in the current case report are available from the corresponding author on reasonable request.
